# Youth-Friendly Sexual Health Services and Peer Support for Improved Sexual and Reproductive Health Outcomes Among Adolescents and Young Adults in South Africa: Results of a Factorial Randomized Controlled Trial

**DOI:** 10.1097/OLQ.0000000000002203

**Published:** 2025-07-21

**Authors:** Jana Jarolimova, Jacob Busang, Natsayi Chimbindi, Nonhlanhla Okesola, Theresa Smit, Guy Harling, Nuala McGrath, Andrew Copas, Janet Seeley, Kathy Baisley, Maryam Shahmanesh, the Isisekelo Research Group, Carina Herbst

**Affiliations:** From the ∗ Massachusetts General Hospital, Boston, MA; † Africa Health Research Institute, Durban, South Africa; ‡ University College London, London, United Kingdom; § University of KwaZulu-Natal, Durban; ¶ University of the Witwatersrand, Johannesburg, South Africa; ∥ University of Southampton, Southampton; ∗∗ London School of Hygiene & Tropical Medicine, London, United Kingdom

## Abstract

**Background:**

Adolescents and young adults in South Africa have high burdens of sexually transmitted infections (STIs) and unintended pregnancy. We evaluated the impact of peer support and/or expanded sexual and reproductive health (SRH) services on STIs, contraception, and pregnancy in rural KwaZulu-Natal, South Africa.

**Methods:**

We analyzed secondary outcomes from a 2 × 2 factorial randomized controlled trial conducted from March 2020 to August 2022 among 16- to 29-year-olds, comparing (1) enhanced standard of care (SoC), access to mobile youth-friendly HIV prevention (AYFS); (2) SRH, self-collected STI testing and referral to AYFS with expanded SRH services; (3) peer support, peer navigator facilitation of AYFS attendance; (4) SRH + peer support. At 12 months, all participants were offered STI testing; female participants self-reported contraceptive use and pregnancy.

**Results:**

Among 1743 trial participants (51% female), 927 (53%) had 12-month STI results; 209 (22.5%) tested positive: 163 (17.6%) chlamydia, 54 (5.8%) gonorrhea, and 44 (4.8%) trichomoniasis. The prevalence of STI was somewhat lower among those exposed to peer support (adjusted odds ratio [aOR] adjusted for sex, age, location, 0.77; 95% confidence interval, 0.56–1.06) or SRH (aOR, 0.74; 0.56–1.06) and, compared with SoC, was reduced in those exposed to both (aOR, 0.59; 0.38–0.94). In SRH arms, 64 of 469 (13.6%) had a new STI at 12 months, with no difference by peer support (*P* = 0.97). Among females, 336 of 634 (53.0%) reported using contraception and 47 of 667 (7.1%) reported pregnancy, with little difference by study arm.

**Conclusions:**

Peer support and STI testing with expanded SRH each had no more than small effects on STIs, contraception, or pregnancy. Combined or more intensive interventions, for example, repeat screening, enhanced partner notification, and deeper understanding of structural drivers, are needed.

## Abstract 2

Peer support and integrated sexual and reproductive health each had only small effects on sexually transmitted infections, contraceptive use, and pregnancy among young people in rural South Africa; combined or more intensive interventions are needed.

Adolescents and young adults (AYA) in South Africa experience a high burden of poor sexual and reproductive health (SRH) outcomes. Greater than 40% of the 1 million new daily global infections with gonorrhea, chlamydia, trichomoniasis, and syphilis occur in sub-Saharan Africa.^1^ In South Africa specifically, sexually transmitted infection (STI) prevalence rates are estimated to be as high as 14.7% among adult women and 6.0% among men for chlamydia, and 6.6% among women and 3.5% among men for gonorrhea,^2^ and are highest in youth aged 15 to 24 years.^3,4^ In South Africa, STIs are syndemic with HIV, increasing the risks of HIV transmission and acquisition when untreated and sharing common risk factors.^5,6^ In the province of KwaZulu-Natal, population-based studies have shown curable STI prevalence as high as 20% among young women and 10% among young men.^3,4^ This same age group also experiences a high rate of unintended and teenage pregnancies and unmet contraceptive need.^7^

Untreated curable STIs can lead to substantial morbidity, particularly for women, including pelvic inflammatory disease, tubal infertility, and pregnancy complications.^8–11^ However, multiple barriers limit young people's attainment of optimal SRH outcomes. Diagnosis and management of STI is limited by a lack of accessible and affordable diagnostic testing, leading to reliance on syndromic management and absence of asymptomatic screening for high-risk populations.^12^ This results in the undertreatment of STIs, most of which are asymptomatic, particularly in women.^13,14^ Access to comprehensive SRH care in clinics and other health care facilities for young adults is limited by stigma, lack of privacy, judgmental approaches from health care workers, and logistical barriers.^15,16^ The World Health Organization, in the 2022–2030 Global Health Sector Strategic Plan on HIV, Viral Hepatitis, and Sexually Transmitted Infections, thus recommends both an expansion in diagnostic testing and screening for STIs and the development of age-appropriate and positive sexual health education and services.^17^

There is growing evidence on the effectiveness of integrated, community-based SRH services. A population-based study in KwaZulu-Natal found that home-based STI specimen collection was highly acceptable among AYA,^4^ and mobile-clinic based STI testing has found high rates of untreated STIs in rural South Africa.^18^ Community-based STI testing could both decrease the prevalence and complications of curable STIs and create demand for further SRH services. In some settings, STI diagnosis has been associated with subsequent uptake of HIV preexposure prophylaxis (PrEP).^19^ Furthermore, there is growing evidence for peer-led interventions to support HIV prevention,^20^ and peer navigation and support could similarly enable linkages to comprehensive SRH care including STI testing. We hypothesized that integrated SRH services including home-based self-sampling for STI testing and referral to youth-friendly clinics, along with peer support, could improve SRH outcomes in rural South Africa, including STI positivity, pregnancy, and contraceptive use at 12 months.

## METHODS

### Study Design

This secondary outcome analysis reports results from a 2 × 2 factorial randomized controlled trial (Isisekelo Sempilo, NCT 04532307) evaluating acceptability, feasibility, and preliminary population-level impact of peer navigator support, with or without comprehensive SRH services, on the prevalence of transmissible HIV. The 4-arm trial enrolled AYA aged 16 to 29 years in uMkhanyakude district in rural KwaZulu-Natal, South Africa. Full trial details are described in the protocol paper.^21^

### Study Setting

The study was conducted within a health and demographic surveillance site (HDSS), where the Africa Health Research Institute has been conducting annual household-based surveys since 2000. The HDSS covers 845 km^2^ with ~140,000 individuals in 20,000 households, including more than 20,000 AYA aged 16 to 29 years.^22^ The mostly rural study area has high unemployment (62% of adults without formal employment) and HIV prevalence of 19% among men and 40% among women aged 15 to 54 years.^22^

### Study Procedures

Using the HDSS as a sampling frame, 3000 AYA, stratified by sex and area, were randomly selected to be assessed for eligibility. Men and women aged 16 to 29 years, residing in the HDSS area, willing and able to provide informed consent, and willing to be contacted at 12 months, were eligible. At enrollment, participants were randomized between 4 study arms (Fig. S1, http://links.lww.com/OLQ/B244): (*a*) enhanced standard of care (SoC; referral to adolescent and youth-friendly services [AYFS] comprising clinic-based, nurse-led HIV-focused services), (*b*) SRH (home-based self-collection of STI specimens with referral to AYFS for STI results management with integrated, expanded SRH services), (*c*) peer support (referral to peer navigator to assess social, health, and educational needs and provide risk-informed HIV prevention and referral to AYFS),^23^ or (*d*) combined SRH and peer support. Participants randomized to the 2 SRH arms were offered home-based STI self-sampling at enrollment, as well as expanded counseling and service provision related to sexual health, fertility, and family planning, provided through the AYFS, beyond that offered in the SoC.^21^ For the STI testing, participants were instructed to self-collect vaginal swab or urine specimens during a study staff home visit, and were given an invitation card to attend the AYFS after 7 days to receive SRH services and the test results. Participants with positive STI results who did not present to the AYFS clinics within 7 days were contacted individually for treatment. In the peer support arms, participants were offered support of named peer navigators residing in their area, trained to provide one-to-one HIV prevention counseling, health promotion, and support in accessing clinical services. Peer support activities, which normally occur in person, were temporarily transitioned to virtual support from March 24, 2020, to November 24, 2020, because of the COVID-19 pandemic.

Adolescent and youth-friendly services, the enhanced SoC upon which the other interventions were layered, were gender-neutral and HIV status–neutral HIV prevention and treatment services and basic primary care SRH services delivered by study nurses in 2 primary health clinics and 2 mobile clinics that visited fixed sites across the surveillance area every 2 weeks. For participants in the 2 SRH arms, expanded SRH services to be provided by the AYFS were emphasized at the time of referral. All AYFS clinic attendees were offered HIV counseling and testing, with immediate ART initiation if found to be living with HIV, or PrEP if negative and eligible (i.e., HIV negative on rapid antibody testing, no symptoms of acute HIV, willing and able to take PrEP as prescribed, no contraindications to oral PrEP, ≥35 kg^24^). Furthermore, all clinic attendees were offered counseling around U = U (undetectable = untransmittable); pregnancy testing and basic family planning support; syphilis, hepatitis B testing, and vaccines; and STI syndromic management. Participants were encouraged to attend the service 3 monthly for HIV testing, PrEP and contraception refills and SRH services. Attendance at AYFS services was recorded for all enrolled participants; initial visits were tracked with a barcoded referral slip.

All participants were contacted 12 months after enrollment. Participants completed a questionnaire that included uptake and experience of HIV services through the AYFS, use of contraception, and pregnancy incidence (among females), and were offered STI testing by home-based self-collection. For female participants, research staff described the procedure to self-collect a vaginal swab. Menstruating females provided urine specimens. Male participants were instructed to collect a first-catch urine specimen. All STI specimens collected either at enrollment (in the 2 SRH arms) or during the 12-month survey were transported to the Africa Health Research Institute central laboratory in Durban, where testing for *Neisseria gonorrhoeae*, *Chlamydia trachomatis*, and *Trichomonas vaginalis* was conducted by real-time polymerase chain reaction using GeneXpert (Cepheid, Sunnyvale, CA). Participants with positive results were contacted for treatment according to South African national guidelines and counseled on partner notification.^25^

### Measures

Valid STI test results were recorded as “detected” or “not detected,” or as “invalid” or “error” based on test output. New STI was defined as testing positive for 1 of the 3 STIs at 12 months after testing negative for the same STI at baseline. Use of contraception at 12 months was assessed by the question, “Are you currently using any contraceptive methods to prevent pregnancy?” New pregnancy was assessed by the question, “Are you currently pregnant?” Sociodemographic data, including education (whether still in school, years completed) and current employment (employed, not employed, studying), were obtained from the 12-month survey. Residence (urban/peri-urban, rural) was derived from linking study participants to the HDSS household-level survey.

### Statistical Analysis

We summarized demographic data using medians and interquartile ranges for continuous variables and frequency counts and percentages for categorical variables. We fitted logistic regression models to jointly estimate the odds ratio (OR) and 95% confidence interval (CI) for the main effects of peer navigator support and the SRH intervention on STI prevalence, contraceptive use, and pregnancy at 12 months, assuming no interaction, in an intention-to-treat analysis. As a secondary approach, we also fitted a 4-level categorical variable to estimate the effect of each trial arm, that is, peer support alone, SRH alone, and peer support combined with SRH, all relative to SoC. We additionally conducted a per-protocol adjusted main effects analysis limited to those participants who attended at least one AYFS clinic visit. We tested for an interaction between peer support and SRH interventions on 12-month STI positivity. We compared new STIs at 12 months between the SRH arms using a *χ*^2^ test. Characteristics of participants who were reached for the endline survey and consented to STI testing were compared with all enrolled participants using univariate and multivariable logistic regressions to assess for any differences. Missing data were not imputed, and participants with missing data were not included in multivariable analyses. All reported *P* values were 2-tailed; *P* < 0.05 was considered statistically significant. Analyses were conducted using Stata version 16.1 (Stata Corp, College Station, TX).

### Role of the Funding Source

The funder had no role in study design, data collection, data analysis, data interpretation, or writing of the report. All authors had full access to all study data and accept responsibility for the decision to submit for publication.

## RESULTS

**Figure 1:**
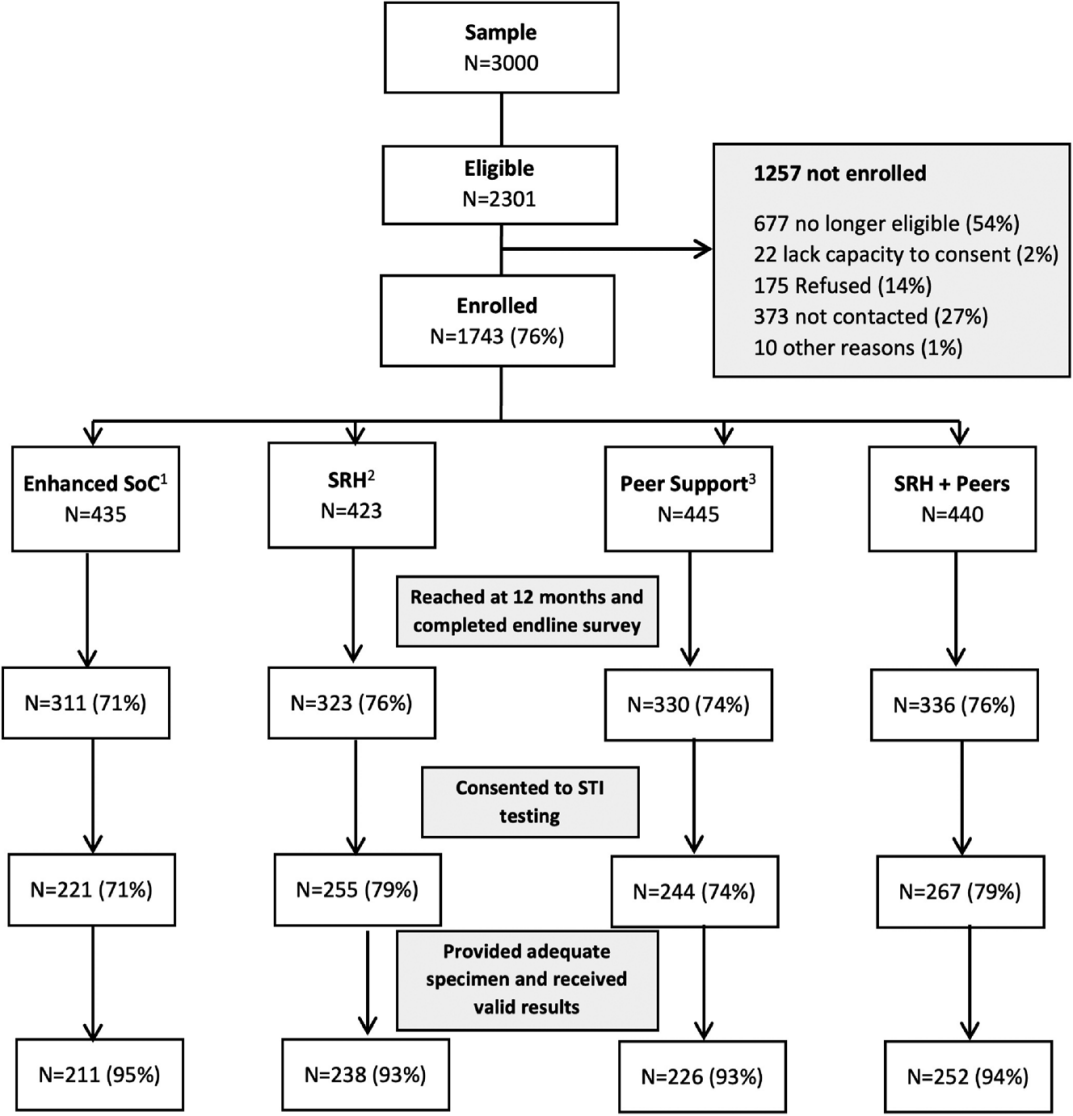
CONSORT diagram.^1^Enhanced SoC—AYFS.^2^Sexual and reproductive health component.^3^Peer navigator component.

Between March 2, 2020, and May 18, 2021, 2627 (88%) of the 3000 AYA sampled were contacted, among whom 2301 (88%) were eligible and 1743 (76%) consented to enrollment (Fig. 1). Primary outcomes of the trial, which showed that the SRH intervention increased linkage to AYFS but neither intervention reduced transmissible HIV, have been previously reported.^26^ There were no important differences in baseline characteristics by arm. Within 60 days of enrollment, 755 participants (43%) linked to clinical services and attended at least one AYFS visit; significantly higher in the SRH study arms.^26^ Overall, 519 (29.8%) of 1743 participants attended AYFS more than once; this was highest in the combined SRH and peer support arm (34%).^26,27^

Among the 1743 enrolled participants, 1300 (75%) were reached at 12 months for the endline survey, of whom 987 (76%) consented to STI testing. In adjusted analyses, those reached at 12 months and consented to STI testing were more likely to have been randomized to one of the SRH arms, to be female, to reside in a rural area, and to be unemployed (Table S1, http://links.lww.com/OLQ/B244). In total, 927 of 987 (94%) participants provided adequate STI specimens and received valid test results and thus contributed data to analyses of 12-month STI outcomes. Contraceptive use and pregnancy data in the 12-month survey were available for 634 of 687 (92%) and 667 of 687 (98%) female participants, respectively.

**Figure 2:**
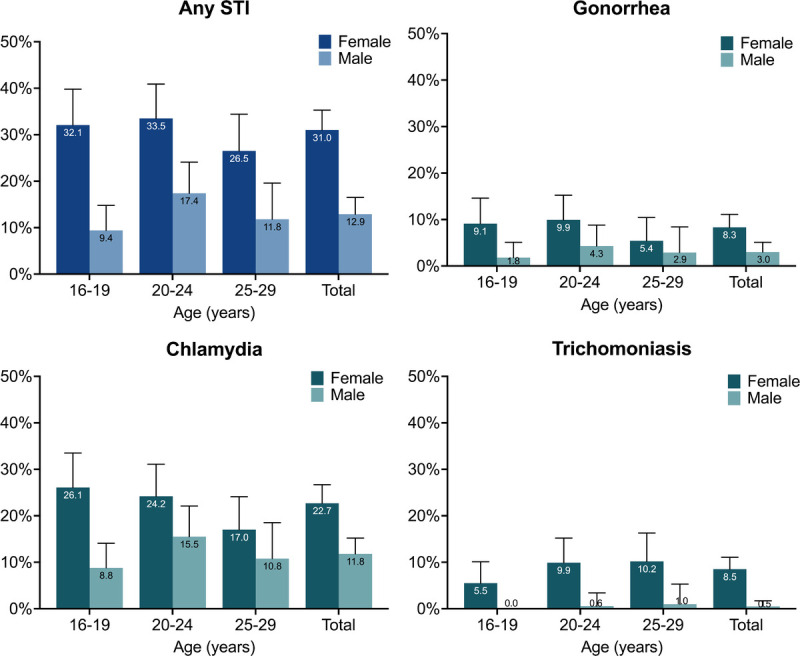
Sexually transmitted infection positivity at 12 months (n = 927).

**Figure 3:**
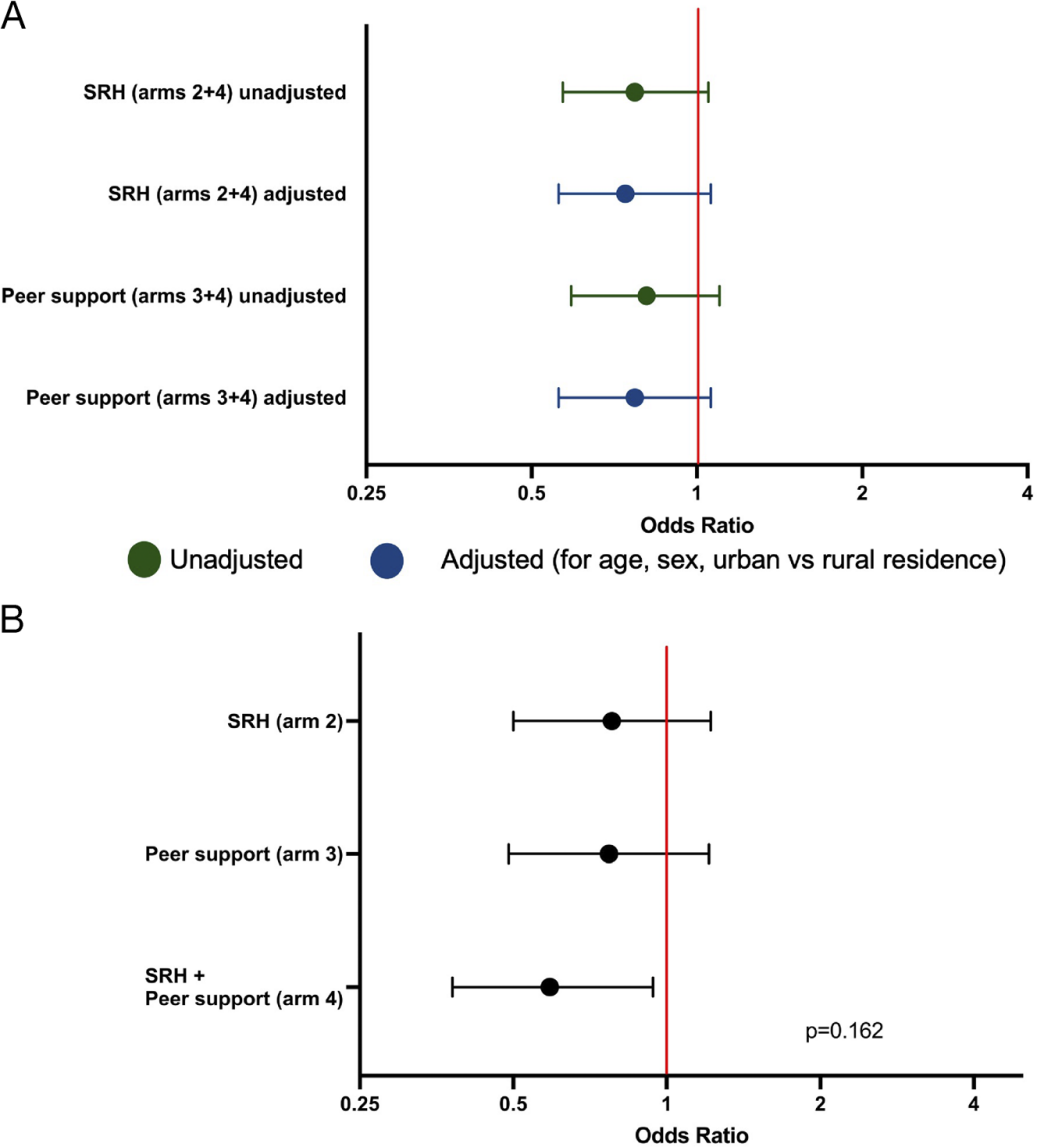
Sexually transmitted infection positivity at 12 months by trial interventions, compared with nonreceipt of each intervention (A) and by trial arm, compared with enhanced SoC (arm 1), adjusted for age, sex, and urban versus rural residence) (B).

Among the 927 participants with complete 12-month STI results, 209 (22.5%) tested positive for at least 1 STI: 163 (17.6%) chlamydia, 54 (5.8%) gonorrhea, and 44 (4.8%) trichomoniasis (Table 1). Positivity for STI by sex and age is shown in Figure 2. In the primary main effects analysis, adjusting for sex, age, and location, 12-month STI positivity was somewhat lower in the SRH intervention arms compared with non-SRH arms (20.4% vs. 24.9%; adjusted OR [aOR], 0.74; 95% CI, 0.56–1.06) and in peer support arms (20.7% vs. 24.5%; aOR, 0.77; 95% CI, 0.56–1.06; Table 1, Fig. 3). There was little evidence of interaction between peer support and SRH (*P* = 0.978). There was not clear variability in STI positivity between the 4 trial arms in a “global” test (*P* = 0.16) after adjustment. However, when compared with SoC, there was some evidence of lower STI positivity, particularly in the combined SRH and peer support arm (aOR, 0.59; 95% CI, 0.38–0.94; Table 1, Fig. 3). In a per-protocol adjusted main effects analysis limited to participants who attended at least one AYFS visit (n = 856), 12-month STI positivity was lower among those assigned to SRH intervention arms (20.4% vs. 27.6%; aOR, 0.69; 95% CI, 0.50–0.96; *P* = 0.026) and those assigned to peer support arms (20.9% vs. 26.7%; aOR, 0.70; 95% CI, 0.50–0.97; *P* = 0.030; Table S2, http://links.lww.com/OLQ/B244).

**TABLE 1:** Prevalence of Any STI, Chlamydia, Gonorrhea, and Trichomoniasis at 12 Months (N = 927)*

	Number With Outcome/Total (%)	Unadjusted OR (95% CI)	*P*	Adjusted OR^†^ (95% CI)	*P*
Prevalence of any STI at 12 mo					
Overall	209/927 (22.5)				
SRH^‡^			*P* = 0.100		*P* = 0.115
No	109/437 (24.9)	1		1	
Yes	100/490 (20.4)	0.77 (0.57–1.05)		0.74 (0.56–1.06)	
Peer support			*P* = 0.168		*P* = 0.104
No	110/449 (24.5)	1		1	
Yes	99/478 (20.7)	0.81 (0.59–1.10)		0.77 (0.56–1.06)	
Trial arm			*P* = 0.200		*P* = 0.162
Enhanced SoC^§^	57/211 (27.0)	1		1	
SRH	53/238 (22.3)	0.77 (0.50–1.19)		0.78 (0.50–1.22)	
Peer support	52/226 (23.0)	0.81 (0.52–1.25)		0.77 (0.49–1.21)	
SRH + peer support	47/252 (18.7)	0.62 (0.40–0.96)		0.59 (0.38–0.94)	
Prevalence of chlamydia at 12 mo					
Overall	163/927 (17.6)				
SRH^‡^			*P* = 0.080		*P* = 0.099
No	87/437 (19.9)	1		1	
Yes	76/490 (15.5)	0.74 (0.53–1.04)		0.75 (0.53–1.06)	
Peer support			*P* = 0.165		*P* = 0.116
No	87/449 (19.4)	1		1	
Yes	76/478 (15.9)	0.79 (0.56–1.10)		0.76 (0.54–1.07)	
Trial arm			*P* = 0.157		*P* = 0.145
Enhanced SoC	45/211 (21.3)	1		1	
SRH	42/238 (17.7)	0.79 (0.49–1.26)		0.80 (0.50–1.29)	
Peer support	42/226 (18.6)	0.84 (0.53–1.35)		0.81 (0.50–1.31)	
SRH + peer support	34/252 (13.5)	0.58 (0.35–0.94)		0.56 (0.34–0.92)	
Prevalence of gonorrhea at 12 mo					
Overall	54/927 (5.8)				
SRH			*P* = 0.204		*P* = 0.256
No	30/437 (6.9)	1		1	
Yes	24/490 (4.9)	0.70 (0.40–1.21)		0.72 (0.41–1.27)	
Peer support			*P* = 0.813		*P* = 0.723
No	27/449 (6.0)	1		1	
Yes	27/478 (5.7)	0.94 (0.54–1.62)		0.90 (0.52–1.58)	
Trial arm			*P* = 0.640		*P* = 0.698
Enhanced SoC	15/211 (7.1)	1		1	
SRH	12/238 (5.0)	0.69 (0.32–1.52)		0.71 (0.32–1.56)	
Peer support	15/226 (6.6)	0.93 (0.44–1.95)		0.89 (0.42–1.88)	
SRH + peer support	12/252 (4.8)	0.65 (0.44–1.43)		0.65 (0.30–1.44)	
Prevalence of trichomoniasis at 12 mo					
Overall	44/927 (4.8)				
SRH			*P* = 0.697		*P* = 0.598
No	22/437 (5.0)	1		1	
Yes	22/490 (4.5)	0.89 (0.48–1.62)		0.85 (0.45–1.58)	
Peer support			*P* = 0.407		*P* = 0.353
No	24/449 (5.4)	1		1	
Yes	20/478 (4.2)	0.77 (0.42–1.42)		0.74 (0.40–1.39)	
Trial arm			*P* = 0.740		*P* = 0.724
Enhanced SoC	13/211 (6.2)	1		1	
SRH	11/238 (4.6)	0.74 (0.32–1.68)		0.75 (0.32–1.74)	
Peer support	9/226 (4.0)	0.63 (0.26–1.51)		0.65 (0.26–1.58)	
SRH + peer support	11/252 (4.4)	0.70 (0.30–1.59)		0.64 (0.28–1.50)	

*Includes data from participants who provided STI specimens and had valid test results for all 3 STIs during the 12-month survey.

^†^Adjusted for sex, age group, and location of residence.

^‡^Adolescent and youth friendly SRH services including home-based STI self-sampling.

^§^Enhanced standard of care.

Among the 469 participants with both baseline and endline STI test results, 64 (13.6%) acquired a new STI during the 12-month study period; this rate did not differ by assignment to the peer support intervention (Table 2). Thirty-nine participants (8.3%) tested positive for the same STI at baseline and endline, demonstrating either persistent infection or re-infection; this rate also did not differ by assignment to peer support (Table 2; Table S3, http://links.lww.com/OLQ/B244).

**TABLE 2:** New Sexually Transmitted Infections (STIs) at 12 Months (N = 469)*

	Overall (N = 469)	SRH^†^ (N = 226)	SRH + Peer Support (N = 243)	*P* ^‡^
Any STI at baseline, n (%)	111 (23.7)	55 (24.3)	56 (23.1)	0.742
Any STI at endline, n (%)	95 (20.3)	49 (21.7)	46 (18.9)	0.459
Any new STI, n (%)^§^	64 (13.6)	31 (13.7)	33 (13.7)	0.966
Diagnosed with same STI at endline as at baseline, n (%)	39 (8.3)	21 (9.3)	18 (7.4)	0.460

*Includes data from participants with valid STI results at baseline and at 12 months (limited to participants in the IS/SRH arms only).

^†^Adolescent and youth friendly SRH services including home-based STI self-sampling.

^‡^*χ*^2^ Test of independence.

^§^Participants who tested negative for any individual STI at baseline and positive for that STI at 12 months.

At study end, 336 of 634 (53.0%) female participants reported using any method of contraception, with no difference by SRH (*P* = 0.301) or peer support intervention (*P* = 0.797; Table S4, http://links.lww.com/OLQ/B244). In a per-protocol analysis limited to female participants who attended at least one AYFS clinic visit (n = 428) and adjusted for age and urban versus rural residence, report of any contraceptive use at the end of the study did not differ by assignment to the SRH (*P* = 0.830) or peer navigator intervention (*P* = 0.798; Table S5, http://links.lww.com/OLQ/B244). At the same time point, 47 of 667 (7.1%; 95% CI, 5.2%–9.3%) female participants reported being pregnant; among women aged 16 to 19 years, 14 of 237 (5.9%; 95% CI, 3.3%–9.7%) reported being pregnant (Table S6, http://links.lww.com/OLQ/B244). Pregnancy did not differ by assignment to the peer navigator intervention (*P* = 0.67), but there was some evidence of fewer pregnancies under assignment to the SRH intervention (*P* = 0.064; Tables 3 and 4; Table S7, http://links.lww.com/OLQ/B244).

**TABLE 3:** Contraception Use Among All Females at the End of the Study (N = 634)

	No. Self-Reporting Contraception Use/Total (%)	Unadjusted OR (95% CI)	Adjusted OR* (95% CI)
Overall	336/634 (53.0)		
SRH^†^		*P* = 0.152	*P* = 0.301
No	150/300 (50.0)	1	1
Yes	186/148 (44.3)	1.26 (0.92–1.72)	1.19 (0.86–1.65)
Peer support		*P* = 0.940	*P* = 0.797
No	167/316 (52.9)	1	1
Yes	169/318 (53.1)	1.01 (0.74–1.38)	1.04 (0.75–1.45)
Trial arm		*P* = 0.560	*P* = 0.716
Enhanced SoC^‡^	76/152 (50.0)	1	1
SRH	91/164 (55.5)	1.25 (0.80–1.94)	1.29 (0.81–2.05)
Peer support	74/148 (50.0)	1.00 (0.64–1.57)	1.13 (0.70–1.82)
SRH + peer support	95/170 (44.1)	1.27 (0.82–1.96)	1.24 (0.78–1.97)

*Adjusted for, age group, and location of residence.

^†^Adolescent and youth friendly SRH services including home-based STI self-sampling.

^‡^Enhanced standard of care.

**TABLE 4:** Female Participants Who Self-Reported Pregnancy at the End of the Study (N = 667)*

	No. Self-Reporting Contraception Use/Total (%)	Unadjusted OR (95% CI)	Adjusted OR^†^ (95% CI)
Overall	47/667 (7.1)		
SRH^‡^		*P* = 0.093	*P* = 0.064
No	28/318 (8.8)	1	1
Yes	19/349 (5.4)	0.60 (0.33–1.09)	0.57 (0.31–1.04)
Peer support		*P* = 0.689	*P* = 0.668
No	22/331 (6.7)	1	1
Yes	25/336 (7.4)	1.13 (0.62–2.05)	1.14 (0.63–2.07)
Trial arm		*P* = 0.560	*P* = 0.297
Enhanced SoC^§^	13/159 (8.2)	1	1
SRH	9/172 (5.2)	0.62 (0.26–1.49)	0.61 (0.25–1.46)
Peer support	15/159 (9.4)	1.17 (0.54–2.55)	1.22 (0.56–2.67)
SRH + peer support	10/177 (5.7)	0.67 (0.29–1.58)	0.65 (0.27–1.52)

*Includes data from female participants who completed the 12-month survey and answered “yes” or “no” regarding self-reported pregnancy.

^†^Adjusted for age group, and location of residence.

^‡^SRH: Adolescent and youth friendly SRH services including home-based STI self-sampling.

^§^Enhanced standard of care.

## DISCUSSION

To our knowledge, this is one of the first trials to examine the effectiveness of comprehensive SRH services including home-based self-sampling for STI testing and peer navigator support for STIs and SRH outcomes among a representative sample of AYA in southern Africa. We did not find clear evidence that either access to home-based, self-collected STI screening with expanded SRH services or peer support individually reduced curable STIs or pregnancy, or increased contraception use, after 12 months of follow-up when compared with an enhanced SoC. However, we did find some evidence of a potential “additive” effect of the SRH and peer support interventions in combination to reduce prevalent STIs, and an effect for both interventions individually among those participants who did attend youth-friendly clinic services during the study period.

We found high STI prevalence in this cohort, both at baseline (22.4%)^28^ and at 12 months (22.5%). Among participants with STI results at both time points, 13.6% acquired a new STI during the study period. Although 12-month STI prevalence did not clearly differ by assignment to either intervention, there was evidence of a potential additive effect (additive on the log-odds scale, without interaction) when interventions were combined. In addition, in a per-protocol analysis limited only to participants who attended at least one AYFS clinic visit during the 12-month study period, both the SRH and peer support interventions were significantly associated with a lower 12-month STI prevalence. The primary outcomes analysis of the trial demonstrated that combined peer support and SRH interventions increased retention in AYFS services.^26^ This suggests that the 2 interventions may bolster the effect of the AYFS services on STI risk, in part by improving retention in services, but the effect of each intervention on its own, without associated and ongoing connection to services, may be modest. Combined and more intensive approaches may thus be needed in this setting, such as repeat STI testing, point-of-care testing (POCT), test of cure, and enhanced partner services including expedited treatment. Furthermore, structural drivers such as poverty, gender inequality, and lack of health care access likely play a significant role in the high STI prevalence in this population.^29^ Thus, conceptually different approaches are likely also needed to impact STI rates, such as a focus on broader social determinants of sexual health behaviors, focus on the efficacy of counseling delivered, and evaluation of ways to enhance engagement, uptake, and continuity of attendance to supplement interventions and maximize efficacy and reach.

Among participants found to have the same STI at baseline and 12 months, most were treated after their baseline testing, suggesting a high rate of reinfections. Screening for curable STIs needs to be incorporated into a broader intervention and delivery mechanism, with effective tools to prevent subsequent reinfection. Partner services, including partner notification, assisted partner services, and expedited partner therapy, are all aimed at decreasing STI reinfection.^30^ However, uptake of partner notification has been low among AYA in sub-Saharan Africa, and efforts to strengthen partner notification services have not consistently led to decreased incidence rates.^30^ Additional strategies, such as improving the acceptability of partner notification services, and enhanced approaches, such as expedited partner therapy, may be needed to strengthen the impact of screening and treatment.

Among those testing positive for STIs at baseline, greater than 40% were unable to be treated within 4 weeks of specimen collection,^28^ primarily due to difficulty contacting participants. High rates of incomplete or delayed STI treatment were also found in a study in Zimbabwe providing community-based STI testing to AYA.^31^ These findings highlight the need for POCTs to enable treatment within the same clinic- or community-based encounter. Development of POCTs that meet WHO REASSURED criteria is a priority for STI control worldwide.^32^ Point-of-care tests could both optimize rates of STI treatment and expand STI testing capacity outside of clinical settings to increase access. Accessible, cost-effective tests could also enable more frequent STI screening, in line with recommendations for repeated STI screening for individuals at increased risk^33^ and findings that frequent screening is likely needed to lower prevalence.^34^

We found high uptake of home-based STI specimen self-collection among this cohort. Moreover, those who had been offered home-based testing at baseline were more likely to accept it at study end. In the clinical trial, the SRH intervention increased linkage to AYFS and retention in risk-differentiated HIV prevention and care.^26^ These results suggest that STI testing is acceptable and has the potential to increase awareness and engagement in STI, SRH, and HIV care. Sexual and reproductive health services including STI testing and treatment may thus serve as an acceptable pathway to HIV testing and risk-differentiated prevention for AYA in these settings. They may also be a mechanism for prevention of reinfection.

We additionally found that rates of self-reported contraceptive use and pregnancy at 12 months did not differ by study arm. This lack of difference may reflect the strength of the “enhanced SoC,” as the accessibility and acceptability of the AYFS may have led to similar exposure to family planning education and access across study arms, irrespective of randomization. The findings may also reflect the fact that this age group encompasses the most common age for childbirth in this society and the balance between STI prevention and pregnancy intentions may need to be balanced. Overall rates of reported contraceptive use were similar to those estimated nationally for South Africa.^35^ The teenage pregnancy rate of 5.9% overall was lower than the data from this setting previously (11%; unpublished data), suggesting that accessible SRH services may be a useful tool in reducing the persistently high rates of teenage and unintended pregnancy in South Africa.

This study has several limitations. More than half of trial participants could be followed up and provided consent for STI testing at 12 months and thus included in our analyses. Rates of new or repeat STIs could only be determined for participants randomized to study arms with baseline STI testing; these numbers also limited our power to detect a difference in STI acquisition rates by peer navigator support. Participants randomized to SRH study arms and females were more likely to accept STI testing at 12 months, potentially introducing bias in the 12-month STI prevalence rates. The small absolute number of reported pregnancies at study end may have limited our power to detect a difference in pregnancy rate by arm; furthermore, self-reported pregnancy at study end would have missed new pregnancies during the study period that terminated or came to term before the 12-month endline. Confining pregnancy measures to females furthermore provides little insight into impacts on male reproductive behavior. Finally, the study period spanned the highest level of lockdowns due to COVID-19 (beginning late March 2020), when study activities paused and peer support moved to a virtual format; the subsequent process evaluation suggested that young people experienced limitations in phone and data access during this time.^27^ Peer navigator support then remained virtual after the remainder of study activities resumed, affecting the fidelity of the peer support intervention and potentially limiting the ability to detect an impact of this intervention.

In conclusion, we found a high prevalence of STIs and new infections for 12 months in a cohort of AYA in South Africa, with no clear effect from exposure to baseline STI testing or peer navigator support, but a potential additive effect of the 2 interventions on 12-month STI positivity. Similarly, there was no difference between arms in use of contraception or reported pregnancy. Although the ability to detect differences between arms may have been impacted by the enhanced SoC available to all and changes in the peer navigator intervention due to the COVID-19 pandemic, these results suggest that more intensive, combination interventions may impact STI and other SRH outcomes among AYA in rural South Africa.
